# Perspective—weight loss, obstructive sleep apnea therapy, or both for cardiometabolic risk in obesity-related obstructive sleep apnea?

**DOI:** 10.1093/sleep/zsag116

**Published:** 2026-04-25

**Authors:** Brandon Nokes, Brian S Wojeck, Christopher N Schmickl

**Affiliations:** Department of Medicine, Sleep Medicine Section, VA San Diego Healthcare System, San Diego, CA, United States; Department of Medicine, Division of Pulmonary, Critical Care, and Sleep Medicine, University of California at San Diego, La Jolla, CA, United States; Department of Endocrinology, Yale University, New Haven, CT, United States; Department of Medicine, Division of Pulmonary, Critical Care, and Sleep Medicine, University of California at San Diego, La Jolla, CA, United States

**Keywords:** obstructive sleep apnea, obesity, clinical trials research

## Abstract

The emergence of potent and durable obesity pharmacotherapies has the potential to drastically alter the treatment landscape for comorbid obesity and obstructive sleep apnea. Recent clinical trials examining the impact of weight loss on obstructive sleep apnea have demonstrated marked improvements in both weight and obstructive sleep apnea severity, leading to the first Food and Drug Administration approval of a weight loss medication for obstructive sleep apnea. However, several questions remain unanswered regarding which trial and management strategies will best optimize real-world outcomes for patients with comorbid obesity and obstructive sleep apnea in an equitable manner.

## Introduction

Following the SURMOUNT-OSA trial, the US Food and Drug Administration recently approved the highly potent weight-loss medication tirzepatide for moderate-to-severe obstructive sleep apnea (OSA) in patients with comorbid obesity, reshaping care for a population comprising approximately 10 per cent of US adults [1]. Both obesity and OSA are independently associated with a broad range of symptoms and cardiometabolic risks, yet their relative causal contributions remain incompletely understood [2, 3]. OSA is characterized by recurrent upper airway obstruction during sleep, leading to sleep fragmentation and intermittent hypoxemia [3]. For more than three decades, first-line therapy has been continuous positive airway pressure (CPAP), which is highly efficacious in preventing choking during sleep [3]. However, many patients find CPAP challenging, and average use is only about half the night, limiting real-world effectiveness [4].

About 40%–60% of OSA is attributable to excess body weight, which promotes OSA via several mechanisms, including increased adiposity within pharyngeal structures and neck soft tissue [5, 6]. In SURMOUNT-OSA, once-weekly tirzepatide produced ~20 percent weight loss over 1 year, was generally well tolerated, and led to substantial improvements in symptoms, OSA severity, and cardiometabolic risk—with effects comparable to or exceeding those typically achieved with CPAP under real-world conditions [1, 7]. Importantly, although SURMOUNT-OSA included a trial enrolling participants already treated with CPAP, CPAP was discontinued 1 week prior to all outcome assessments. Thus, tirzepatide was evaluated under highly controlled conditions (including intensive lifestyle and adherence counseling) and compared only with placebo—not with CPAP or combination therapy.

A recent national survey [7] found that roughly half of patients would prefer tirzepatide over CPAP if both were equally effective for OSA and related symptoms and health risks under real-world conditions. Similarly, the majority of respondents viewed combination therapy as acceptable if it provided greater benefits than either therapy alone [7]. Accordingly, a central practical question for patients, clinicians, and payers is whether individuals living with comorbid obesity and OSA (COBOSA) should be treated with tirzepatide, CPAP, or combination therapy.

In this context, the mediation analysis from SURMOUNT-OSA by Malhotra and colleagues, recently published in *Nature Medicine* [8], provides a thoughtful and rigorous attempt to disentangle the relative contributions of tirzepatide-induced reductions in body weight versus OSA severity—as measured by the apnea-hypopnea index (AHI) and sleep-apnea–specific hypoxic burden (SASHB)—to improvements in cardiometabolic outcomes. Applying modern causal mediation methods to the rich SURMOUNT-OSA trial data, the authors report that cardiometabolic improvements were driven predominantly by weight loss, with more modest contributions from reductions in OSA severity limited to high-sensitivity C-reactive protein (hs-CRP), homeostatic model assessment of insulin resistance (HOMA-IR), and triglycerides, and no evidence of mediation by OSA severity alone for other outcomes, including blood pressure.

## Pro: Head-to-Head Trials Comparing CPAP and Tirzepatide Are Necessary

**Figure 1:**
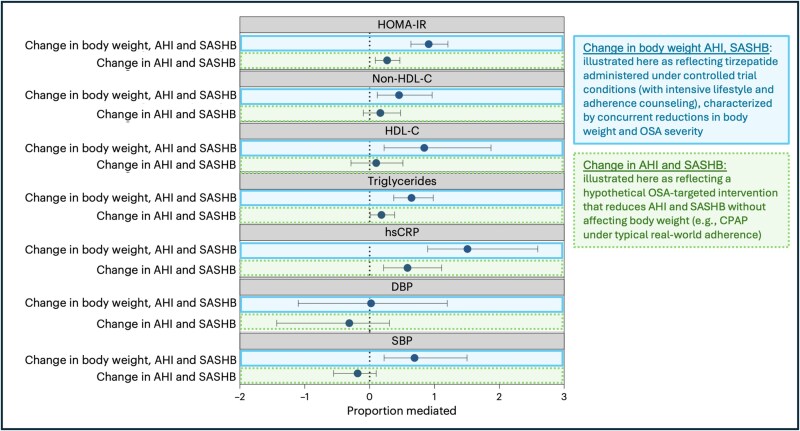
Proportion of tirzepatide’s effect on cardiometabolic risk measures in SURMOUNT-OSA mediated by body weight, apnea hypopnea index (AHI), and sleep-apnea-specific hypoxic burden (SASHB). Adapted from Malhotra et al. [8]. Rows corresponding to “change in AHI and SASHB” and “change in body weight, AHI, and SASHB” are shown to facilitate a conceptual interpretation of the mediation results. This figure illustrates a thought experiment and does not imply causal equivalence or direct comparison between tirzepatide and OSA-specific therapies such as CPAP.

The discussion in the recent article by Malhotra et al. implicitly treats changes in AHI and SASHB as representing the effects of a hypothetical OSA-targeted therapy that improves sleep-disordered breathing without affecting body weight—an interpretation most consistent with the real-world CPAP effects, although not explicitly stated. This framing is intriguing, and in this conceptual thought experiment (Figure 1), tirzepatide under controlled conditions appears non-inferior, and in some cases superior, to expected CPAP effects for the cardiometabolic outcomes examined. However, mediation analyses rely on strong assumptions and are sensitive to model specification. For example, substantial night-to-night variability in AHI introduces measurement error that may attenuate its estimated mediating role, while modeling SASHB as a continuous (log-transformed) variable assumes a smooth relationship with risk, despite evidence for threshold effects at higher values [9]. In addition, tirzepatide’s direct metabolic effects (e.g. on insulin sensitivity) may influence both mediators and outcomes, complicating the assumption of no mediator–outcome confounding. These considerations may help explain the unexpected finding that changes in OSA severity did not mediate blood pressure effects, despite extensive evidence that CPAP lowers blood pressure by 2-3 mmHg in controlled trials. Similarly, mediation proportions exceeding 1 for hsCRP imply a negative direct effect of tirzepatide independent of weight loss and OSA severity—an inference that seems difficult to reconcile biologically and may reflect statistical artifact rather than true antagonism.

While the analysis offers important mechanistic insights, these results—derived from a controlled trial setting—together with the above considerations warrant cautious interpretation and underscore the need for direct, head-to-head comparisons of OSA-targeted (e.g. CPAP) versus weight-loss–based (e.g. tirzepatide) strategies in real-world practice.

Second, because reductions in OSA severity independently mediated tirzepatide’s effects on select outcomes, it is tempting to assume that combining tirzepatide with CPAP would yield additive benefit. However, the confidence intervals for estimates comparing the changes mediated by body weight alone versus body weight plus AHI and SASHB largely overlapped, suggesting limited additional cardiometabolic benefit from OSA improvement within the observed range. Whether further reductions in OSA severity *beyond* those achieved with tirzepatide translate into incremental clinical benefit remains wholly an empirical question. Prior studies comparing lifestyle-induced weight loss, CPAP, and their combination found no significant additive cardiometabolic benefit of CPAP beyond weight loss in intention-to-treat analyses, with modest per-protocol effects susceptible to confounding and healthy-user bias [2]. Moreover, the added cost and treatment impacts of combination therapy may further limit adherence and thus effectiveness.

Third, tirzepatide’s effects are likely heterogeneous, and a “one-size-fits-all” approach risks amplifying health disparities. Treatment response may differ by age, sex, and other patient characteristics, both in terms of weight loss and downstream cardiometabolic benefit [10, 11]. Thus, a careful evaluation of the relative risks and benefits across patient subgroups is needed to ensure equitable care.

In the absence of comparative effectiveness evidence from diverse populations, clinicians, patients, and payers will be forced to make treatment decisions between CPAP, pharmacologic weight loss, and combination strategies without clear guidance regarding relative benefits and treatment impacts. This question has already begun to generate debate in the sleep medicine community, with several recent editorials and commentaries calling for direct head-to-head comparisons between pharmacologic weight-loss therapies and CPAP to guide clinical decision-making [12–14]. However, whether a simple head-to-head trial would be the most informative approach remains debatable.

## Con: Simple Head-to-Head Comparisons of CPAP vs Tirzepatide May Be Insufficient

Direct head-to-head comparisons between CPAP and tirzepatide may not adequately capture the clinical complexity of treating COBOSA. The therapies target different aspects of OSA and are not necessarily substitutes. CPAP provides immediate mechanical stabilization of the upper airway during sleep and remains the most efficacious therapy for eliminating obstructive events, whereas tirzepatide acts upstream by inducing weight loss and modifying metabolic drivers of OSA. Accordingly, in patients with a primary goal of weight reduction, pharmacologic weight-loss therapy is clearly the more appropriate intervention, whereas in those requiring rapid control of OSA symptoms (e.g. safety concerns like drowsy driving), CPAP remains the preferred initial therapy. In these settings, equipoise for a head-to-head trial is lacking.

**Figure 2:**
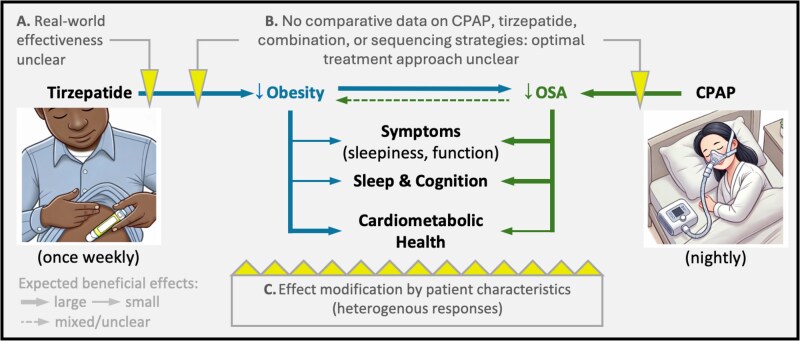
Critical knowledge gaps for the management of patients with comorbid obesity and OSA.

The clinical dilemma arises in patients with COBOSA who do not require urgent control of OSA. In this group, framing CPAP and tirzepatide as competing alternatives risks creating a false dichotomy, as the therapies may be complementary. A trial limited to a two-arm head-to-head comparison would therefore be insufficient; inclusion of a combination arm is essential to determine whether combined therapy provides clinically meaningful incremental benefit relative to either monotherapy, and whether such benefit justifies the added cost and treatment impacts. Notably, a substantial proportion of patients initiating combination therapy may experience sufficient weight loss–related improvement in OSA to discontinue CPAP over time, but the durability of these effects remains uncertain and requires longitudinal evaluation.

Similarly, treatment failure of either CPAP or tirzepatide often becomes apparent within months and in routine care typically leads to treatment modification, including switching or addition of the alternative therapy. Capturing these trajectories—including the need for second-line or rescue therapies such as hypoglossal nerve stimulation—would provide clinically actionable information beyond initial treatment assignment. Sequential trial designs (e.g. SMART) may therefore be better suited to reflect real-world care and inform optimal treatment pathways.

Importantly, there is substantial heterogeneity in OSA pathophysiology. In many individuals, excess weight may represent the dominant or sole driver of disease, and in such patients, head-to-head comparisons of CPAP vs weight-loss–based therapies would be highly informative. However, a substantial number of patients exhibit mixed or non-obesity–dominant mechanisms (e.g. impaired upper airway muscle compensation or ventilatory control instability) [15], in whom weight loss alone may be insufficient. Trials must therefore be designed to assess heterogeneity of treatment effects across clinically relevant subgroups and, ideally, incorporate mechanistic assessments to identify predictors of response.

To be informative for real-world practice, such trials should be pragmatically embedded within routine care and enroll diverse populations. However, access considerations are critical: whereas CPAP is generally covered, access to tirzepatide may be limited, particularly among disadvantaged populations [16, 17]. Trial designs that rely on existing insurance coverage for treatment allocation risk exacerbating disparities.

Ethical considerations do not preclude randomized comparisons, but warrant close attention. Prior consensus statements emphasize that trials can be conducted safely with appropriate safeguards, including careful patient selection, monitoring for adverse outcomes (e.g. excessive sleepiness), and clear informed consent [18].

Finally, the therapeutic landscape is rapidly evolving, with newer incretin-based agents likely to emerge during the course of large-scale trials. Trial designs must therefore be sufficiently flexible to accommodate advances in pharmacotherapy within weight-loss–based treatment strategies.

Taken together, these considerations suggest that trials limited to a static head-to-head comparison may provide incomplete guidance for clinical decision-making. More informative approaches may include combination, sequential, and/or phenotype-guided strategies that better reflect the complexity of real-world care.

## Putting It All Together—A Pragmatic Approach to Real-World Care for Obesity and OSA

The SURMOUNT-OSA mediation analysis provides valuable mechanistic insights while underscoring the inherent limits of inference from secondary analyses, even when rigorously conducted. As tirzepatide and similar medications are increasingly adopted, the central clinical question is no longer whether these interventions work in isolation under ideal conditions, but how they compare—and interact—when deployed in routine care. Importantly, the goals of OSA therapy extend beyond cardiometabolic risk to include symptom impacts—a domain not included in the current mediation analysis but central to patient-centered decision-making.

As clinicians dual-boarded in sleep and obesity medicine, we strongly support a holistic approach that addresses both obesity and OSA, while emphasizing the need for rigorous empirical evaluation of specific treatment strategies. Closing the critical evidence gaps highlighted in Figure 2 will require large, pragmatic comparative effectiveness trials in diverse populations that evaluate CPAP, weight-loss–based, and combination strategies, as well as their sequencing over time under real-world conditions. Such trials should ensure clinical equipoise through careful participant selection, incorporate patient-centered outcomes, include long-term follow up with capture of treatment sequences in patients who do not respond to the initially assigned therapy, and ensure adequate representation of key subgroups. Consideration of access and implementation in routine care settings will be essential to ensure that findings translate equitably into practice.

In parallel, studies clarifying the optimal management of residual OSA following weight loss are needed. As the therapeutic landscape continues to evolve, flexible and adaptive study designs will be important to incorporate emerging pharmacologic options. Such evidence will be essential to optimize care for the millions of individuals living with COBOSA and to guide clinical guidelines, reimbursement decisions, and patient-centered treatment choices.
